# Selumetinib normalizes Ras/MAPK signaling in clinically relevant neurofibromatosis type 1 minipig tissues in vivo

**DOI:** 10.1093/noajnl/vdab020

**Published:** 2021-02-10

**Authors:** Sara H Osum, Alexander W Coutts, Dylan J Duerre, Barbara R Tschida, Mark N Kirstein, James Fisher, W Robert Bell, Oona Delpuech, Paul D Smith, Brigitte C Widemann, Christopher L Moertel, David A Largaespada, Adrienne L Watson

**Affiliations:** 1 Department of Pediatrics, University of Minnesota, Minneapolis, Minnesota, USA; 2 Recombinetics Inc., Eagan, Minnesota, USA; 3 Department of Experimental and Clinical Pharmacology, University of Minnesota, Minneapolis, Minnesota, USA; 4 Division of Neuropathology, Department of Lab Medicine and Pathology, University of Minnesota, Minneapolis, Minnesota, USA; 5 Oncology R&D, AstraZeneca, Cancer Research UK Cambridge Institute, University of Cambridge, Cambridge, UK; 6 Pediatric Oncology Branch, Rare Tumor Initiative, Center for Cancer Research, National Cancer Institute, Bethesda, Maryland, USA

**Keywords:** MEK inhibitor, neurofibromatosis type 1, pharmacodynamics, pharmacokinetics, selumetinib

## Abstract

**Background:**

The MEK1/2 inhibitor selumetinib was recently approved for neurofibromatosis type 1 (NF1)-associated plexiform neurofibromas, but outcomes could be improved and its pharmacodynamic evaluation in other relevant tissues is limited. The aim of this study was to assess selumetinib tissue pharmacokinetics (PK) and pharmacodynamics (PD) using a minipig model of NF1.

**Methods:**

WT (*n* = 8) and NF1 (*n* = 8) minipigs received a single oral dose of 7.3 mg/kg selumetinib. Peripheral blood mononuclear cells (PBMCs), cerebral cortex, optic nerve, sciatic nerve, and skin were collected for PK analysis and PD analysis of extracellular regulated kinase phosphorylation (p-ERK) inhibition and transcript biomarkers (*DUSP6* & *FOS*).

**Results:**

Key selumetinib PK parameters aligned with those observed in human patients. Selumetinib concentrations were higher in CNS tissues from NF1 compared to WT animals. Inhibition of ERK phosphorylation was achieved in PBMCs (mean 60% reduction), skin (95%), and sciatic nerve (64%) from all minipigs, whereas inhibition of ERK phosphorylation in cerebral cortex was detected only in NF1 animals (71%). Basal p-ERK levels were significantly higher in NF1 minipig optic nerve compared to WT and were reduced to WT levels (60%) with selumetinib. Modulation of transcript biomarkers was observed in all tissues.

**Conclusions:**

Selumetinib reduces MAPK signaling in tissues clinically relevant to NF1, effectively normalizing p-ERK to WT levels in optic nerve but resulting in abnormally low levels of p-ERK in the skin. These results suggest that selumetinib exerts activity in NF1-associated CNS tumors by normalizing Ras/MAPK signaling and may explain common MEK inhibitor-associated dermatologic toxicities.

**Key Points**

- Selumetinib plasma PK in minipigs closely models plasma PK in humans.
- Selumetinib normalizes MAPK signaling to WT levels in CNS tissues from NF1 minipigs.
- Selumetinib-associated skin toxicities may be due to over-suppression of MAPK signaling.

**Importance of the Study**

The MEK inhibitor selumetinib was recently approved for inoperable NF1-associated plexiform neurofibromas, but not all patients respond, some exhibit toxicities, and the effectiveness of selumetinib to treat other NF1-associated tumors remains unclear. Due to the need for invasive biopsies and large samples, pharmacokinetic and pharmacodynamic analyses of selumetinib in NF1-relevant tissues using murine models and human patients are limited. We describe a pharmacokinetic and pharmacodynamic analysis of selumetinib in clinically relevant tissues using a minipig disease model of NF1. We demonstrate that selumetinib reaches higher levels in CNS tissues from NF1 animals compared to WT. Further, selumetinib reduces MAPK signaling to WT levels in NF1 optic nerve and white matter tracts in the cerebral cortex, but abolishes MAPK signaling in minipig skin, which could explain common dermatologic toxicities in patients and have implications for future clinical trials with selumetinib and other MEK inhibitors in NF1 patients with central or peripheral nervous system tumors.

Neurofibromatosis type 1 (NF1) predisposes patients to developing tumors of the nervous system.^1–3^ Individuals with NF1 carry loss of one functional allele of the *NF1* gene encoding neurofibromin, a Ras-GTPase-activating protein (Ras-GAP) that functions as a tumor suppressor protein by negatively regulating the Ras-MAPK signaling pathway. Tumors develop after somatic loss of the wild type (WT) *NF1* allele and typically show hyperactive MAPK signaling.^4^ Preclinical and clinical data demonstrate that the MEK1/2 inhibitor selumetinib reduces the size and growth potential of NF1-associated plexiform neurofibromas and low-grade glioma, and selumetinib was recently FDA approved for children with symptomatic inoperable plexiform neurofibroma.^5–8^ However, the ability to assess drug concentration and effect in NF1-relevant tissues has been a challenge. Invasive biopsies are not feasible in human patients and these procedures are difficult in rodent models due to their small size and dissimilar pathobiology. Pigs have proven to be a useful model for human disease given their genetic, anatomic, and physiologic similarities to humans.^9–12^ We developed a gene-edited minipig carrying a germline heterozygous loss-of-function mutation in the *NF1* gene.^13^ This is the first animal model of NF1 to exhibit both café au lait macules and neurofibromas, and initial pharmacology studies in *NF1*^*+/-*^ (NF1) minipigs support their use as a preclinical disease model.^13^ We conducted a preclinical study of selumetinib in clinically relevant tissues from minipigs including blood, skin, and nervous system tissues and showed that selumetinib reduced Ras/MAPK signaling as assessed by both ERK phosphorylation and transcript biomarkers.

## Materials and Methods

### Minipig Generation and Husbandry

To generate a minipig model of NF1, we mimicked a recurrent nonsense mutation p.Arg1947X (R1947X) identified in 62 of 8100 (± 8) unrelated and symptomatic NF1 patients.^14^ This mutation has also been described in several other studies.^15–18^*NF1*^*R1947*^ lies within exon 41 of the swine *NF1* gene, which shares 100% amino acid identity with human exon 39. Transcription activator-like effector nucleases flanking *NF1*^*R1947*^ were transfected into fetal Ossabaw minipig fibroblasts with a homology directed repair oligonucleotide containing the *NF1*^*R1947X*^ mutation and a *Hin*dIII restriction fragment length polymorphism site. Colonies derived from single cells were isolated and genotyped for the *NF1*^*R1947X*^ mutation. Heterozygous clones were subjected to chromatin transfer resulting in 2 viable pregnancies and 8 F0 male piglets. *NF1*^*+/-*^ (NF1) F0 minipigs were sequence validated, subsequently bred to WT sows, and exhibited germline transmission of the mutant *NF1* allele with Mendelian frequency. There was no evidence of reduced fitness in NF1 minipigs, with 105 F1 piglets produced from the first 15 litters: 54% (57) WT and 46% (48) NF1. Germline transmission of the mutant *NF1* allele was also demonstrated by breeding NF1 females to WT males. All animal work was performed in Recombinetics facilities under its Animal Welfare Assurance #A4728-01. All animal protocols were reviewed and approved by the Institutional Animal Care and Use Committee (IACUC) # RCI-1612-10A.

### Vascular Access Port Surgery

Anesthesia was induced with Telazol^®^ (5.5 mg/kg), xylazine (2.75 mg/kg), and ketamine (2.75 mg/kg) by intramuscular injection; then animals were intubated. Anesthesia was maintained with 1%–5% isoflurane and mechanical ventilation with 100% oxygen at 1 L/min/45 kg. Pulse, electrocardiogram, and blood oxygen saturation were monitored continuously and recorded every 5 min. Depth of anesthesia was monitored by corneal reflex and jaw tone. Vascular access port (VAP) implantation was performed essentially as described.^19^ VAPs consisted of a titanium port with a silicone septum and an attachable silicone catheter (Access Technologies, Skokie, Illinois). The port and catheter were flushed with 0.9% saline and locked with 3–5 mL (volume appropriate to the length of catheter) taurolidine-citrate catheter locking solution (TCS) (Access Technologies).

### VAP Maintenance and Access

Maintenance was performed after blood sampling or flushing. The site was prepped with a surgical scrub prior to needle entry using a sterile Huber needle (Access Technologies). After sampling, the port was flushed with 10 mL 0.9% saline and locked with TCS. Ports were maintained for the duration of the study. In the case of occlusion, which was uncommon, a thrombolytic agent (alteplase 1 mg/mL) was infused to fill the volume of the catheter (~3 mL). This was repeated up to 3 times in a 24 h period until the occlusion resolved.

### Drug Formulation and Dosing

Selumetinib was kindly provided by AstraZeneca as a hydrogen sulfate salt and formulated in 10% EtOH, 30% PEG400, 60% Phosal 50 PG to a concentration of 16 mg/mL. Human equivalent doses were determined by allometric scaling using the standard conversion coefficient (*K*_m_), where *K*_m_ = (human weight/minipig weight)^0.75^. Since the minipigs were approximately the same weight, we used an average weight of 40.2 kg to determine *K*_m_. Assuming a typical adult human weight of 70 kg, the *K*_m_ was determined to be 1.5. Consequently, a 450-mg human equivalent dose was 7.4 mg/kg. The required volume of drug for each animal was then based on individual body weight, which was determined on the day of administration.

### Trial Design

#### Dose-finding Study

This was conducted to determine the dose required to reach the human equivalent therapeutic exposure. Human equivalent doses of 50, 75, 300, and 600 mg were tested. Each dose was administered orally to a 5-month-old wild-type female minipig. Blood was collected immediately prior to drug administration (T = 0) and 1, 2, 6, and 24 h after drug administration.

#### Plasma PK and PD Study

WT (*n* = 8) and NF1 (*n* = 8) minipigs received a single human equivalent oral dose of 450 mg (7.3 mg/kg). Blood was collected immediately prior to selumetinib administration (T = 0), and 0.5, 1, 2, 3, 5, 8, 10, 12, 24, and 36 h after selumetinib administration.

#### Tissue PK and PD Study

WT (*n* = 8) and NF1 (*n* = 8) minipigs received a single human equivalent oral dose of 450 mg (7.3 mg/kg). Untreated WT (*n* = 4) and NF1 (*n* = 4) minipigs were also enrolled as controls. All animals were euthanized 2 h after drug administration. Tissue samples were collected from each animal at the same approximate locations and divided for analysis.

Any animals showing abnormal clinical signs or requiring medication within 2 weeks prior to this study were excluded. Animals were enrolled in the study based on random assignment and the order of treatments was also randomly assigned. Animals were given identification numbers (IDs) and the treatment group and genotype were blinded from researchers, whenever possible, until after the data were collected, in order for data analysis and figure compilation.

### PK Analysis

Selumetinib PK studies were performed in plasma, skin, sciatic nerve, optic nerve, and brain. Plasma isolation was performed as described in a previous study.^13^ For tissue PK, animals were euthanized 2 h after Selumetinib administration. Tissues were harvested and immediately transferred to cryovials and stored at −80°C until analysis.

For each tissue type (plasma, skin, sciatic nerve, optic nerve, and cerebral cortex), selumetinib samples were analyzed with liquid chromatography tandem mass spectrometry (LC-MS/MS) based on previously published studies.^20–25^ Reference standards for selumetinib and MEK162 (internal standard) were purchased from Toronto Research Chemicals. Standard curves and controls were generated using corresponding untreated tissue. Skin tissue was treated with collagenase, and all organ tissues were homogenized. Liquid extraction with ethyl acetate was then performed, followed by analysis. Detection and quantification of selumetinib was performed using an HPLC (Agilent 1100 Series) coupled with an API4000 triple quadrupole instrument (MDS-SCIEX). The chromatographic separation was performed as described.^13^ Mass spectrometric detection was performed using multiple reaction monitoring in positive ionization mode. The precursor/product ion pairs monitored were m/z 459–>397 for selumetinib and m/z 442–>380 for the internal standard (MEK162). Ion source gases 1 and 2 were set at 50 and 50 psi, respectively; the curtain gas was at 20 psi and the collision gas at 4 psi. The collision energy was set at 30 eV for selumetinib and 31 eV for the internal standard. Data acquisition was performed with analyst 1.4.1 software (MDS-SCIEX).

Selumetinib plasma concentration–time data were analyzed using noncompartmental methods implemented in R (version 3.6) R Studio PKNCA package (version 0.9.3).^26^ The PK parameters included AUC–time curve from time 0 to ∞ (linear up, log down), C_max_, T_max_, and T_1/2_.

### PD Analysis

PD analysis in blood was performed as described in a previous study, with some modifications.^13^ Whole blood was collected prior to selumetinib administration (T = 0) and 2 and 5 h after selumetinib administration. Samples were stimulated with phorbol 12-myristate 13-acetate (PMA) (200 nM) or phosphate-buffered saline for 10 min at 37°C within 1 h of being drawn. Red blood cells were removed using standard ammonium-chloride-potassium (ACK) lysis. Peripheral blood mononuclear cells (PBMCs) were snap-frozen as dry pellets and stored at −80°C until analysis. Protein lysates from PBMCs were prepared as described in a previous study.^13^ For tissues, protein lysates were prepared by transferring ~30 µg of snap frozen tissue to a 1.7-mL microfuge tube containing radioimmunoprecipitation lysis buffer with protease and phosphatase inhibitors. The samples were then homogenized using a rotor-stator homogenizer for 2–4 cycles of 20 s and centrifuged for 10 min at 4°C. Concentrations were measured using the Pierce BCA Protein assay kit (Thermo #23227), according to the manufacturer’s instructions. A capillary western blot assay was performed on a Wes system (ProteinSimple 004–600) according to the manufacturer’s instructions using a 12–230 kDa Separation Module (ProteinSimple SM-W004) and Anti-Rabbit Detection Module (ProteinSimple DM-001). Lysates were loaded at 1 mg/mL and instrument default settings were used. Primary antibodies against Phospho-p44/42 ERK1/2 (p-ERK) (Cell Signaling Technologies #4370S) and p44/42 ERK1/2 (ERK) (Cell Signaling Technologies #4695S) were used at 1:100 dilution. Peaks of the appropriate molecular weight (42/44 kilodaltons) were detected in the resulting electropherograms and chemiluminescent signal was quantified by calculating the area under the peaks. The relative intensity of p-ERK to total ERK was then calculated as a ratio (p-ERK/ERK). To discriminate low p-ERK signals from background, the peak signal-to-noise (S/N) ratio given by the software was ≥10, and the peak height/baseline ratio was ≥3.^27^

For RNA profiling, tissues were homogenized using ceramic homogenizers (Agilent 5982–9311) and a Beadblaster 24 (Benchmark D2400) for 3 cycles of 30 s each. RNA was extracted from all tissues using the RNeasy Mini kit (QIAGEN 74104) and RNA concentration and quality was measured by NanoDrop (Thermo). Analysis of transcript biomarkers of MEK inhibition was performed using the Fluidigm platform by Reverse Transcription, preamplification with pooled primers and Real Time-PCR on the Juno and BioMark using the following pig TaqMan primers (*DUSP6*-Ss06941845_m1; *FOS* -Ss03390402_m1; normalized to the average of pig housekeeping genes (*B2M*/Ss03391154_m1; *HPRT1*/Ss03388274_m1; *PPIA*/Ss03394782_g1; *RPL13A*/Ss03376908_u1; *UBC*/Ss03374343_g1, *TFRC*/Ss03391240_m1). Data were then normalized to the average of the control untreated group from matching WT or NF1 background (Log_2_ fold change, -ddCt).

### Immunohistochemical Analysis

Formalin-fixed paraffin-embedded minipig tissue including cerebral cortex, cross sections of optic nerve and sciatic nerve, and skin were sectioned. Immunohistochemical staining for phosphor-p44/p42 MAPK (ERK1/2) (Thr202/Tyr204) (Cell Signaling Technology Catalog #4370, Rabbit mAb, 1:100 dilution) was performed using 5 μm sections on a Thermo Fisher Scientific Autostainer 360 (Thermo Fisher Scientific). Immunoreactivity was scored in the highest regional areas demonstrating p-ERK protein expression at 400× magnification localized to Schwann cell or glial cell nuclei in multiple nerve fascicles of the sciatic nerve, optic nerve fibers, or in subcortical white matter regions of cerebral cortex as: no (0), low (1) (1–10 glial cells), moderate (2) (11–20 glial cells), and high (3) (>20 glial cells) in examined tissues. In the skin, epidermis was scored as no (0) or low (1) (<20 epidermal cells per 400× magnification) or high (2) (>20 epidermal cells per 400× magnification) immunoreactivity.

### Statistical Analysis

Linear regression was performed using GraphPad software. All statistical analysis was done using R (version 3.6) or R Studio PKNCA package (version 0.9.3).^26^ Paired or unpaired *t*-tests with or without Welch’s correction were used as indicated in figure legends to generate *P* values with α = 0.05.

## Results

### Dose-finding Study

The maximum tolerated dose of selumetinib in adult patients with advanced solid malignancy is 75 mg twice daily.^28^ In pediatric patients with plexiform neurofibroma or low-grade glioma, the maximum tolerated dose is 25 mg/m^2^ twice daily (approximately 60% of the adult dose).^6,7^ These doses resulted in a median maximum concentration (C_max_) in plasma of 1520 ng/mL and 886 ng/mL, respectively, and a confirmed partial response in 70% of pediatric patients.^6–8^ Based on these data, we performed a pilot study in which juvenile WT minipigs (5 months of age) received a single oral dose of selumetinib and blood was collected over time to estimate plasma pharmacokinetics of selumetinib in minipigs. Human doses of 50, 75, 300, or 600 mg were allometrically scaled to the minipig to obtain a human equivalent, weight-based dose. In this study, selumetinib exposure was not strictly dose proportional, with the 300 mg equivalent dose resulting in a C_max_ of 1000 ng/mL and the 600 mg equivalent dose resulting in a C_max_ of 670 ng/mL (data not shown). However, selumetinib exposure was dose proportional from 75 to 300 mg. To account for individual variability while achieving a C_max_ approximating that seen in human patients, a 450 mg human equivalent dose of 7.3 mg/kg was chosen for subsequent analyses in the minipig.

### Trial Design

**Figure 1.**
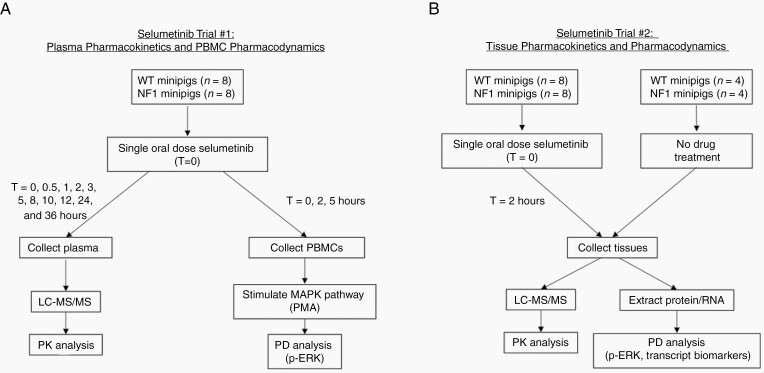
Trial design and analysis of selumetinib pharmacokinetics and pharmacodynamics in minipig blood and tissues. (A) Trial #1 assessed selumetinib plasma pharmacokinetics and PBMC pharmacodynamics over time. (B) Trial #2 assessed selumetinib tissue pharmacokinetics and pharmacodynamics over time. LC-MS/MS, liquid chromatography tandem mass spectrometry; MAPK, mitogen-activated protein kinase; PBMC, peripheral blood mononuclear cell; p-ERK, phosphorylated ERK; PK, pharmacokinetics; PMA, phorbol 12-myristate 13-acetate; WT, wild type.

A single oral dose of 7.3 mg/kg selumetinib was administered to 16 juvenile minipigs (8 WT, 8 NF1). This sample size was chosen to include at least 4 animals of each sex and each genotype and ensure a large enough data set for statistical analyses. Blood was collected prior to drug administration and at 0.5, 1, 2, 3, 5, 8, 10, 12, 24, and 36 h after drug administration for PK analysis and at 0, 2, and 5 h for PD analysis (Figure 1A). For tissue PK and PD analyses, a single oral dose of 7.3 mg/kg selumetinib was administered to the same 16 minipigs after a washout period, and an additional 8 age-matched minipigs (4 WT, 4 NF1) were enrolled as untreated controls. All animals were euthanized 2 h after selumetinib administration and skin, sciatic nerve, optic nerve, and cerebral cortex were collected for tissue PK and PD analysis (Figure 1B). No data points were excluded from this study.

### Blood PK

**Figure 2.**
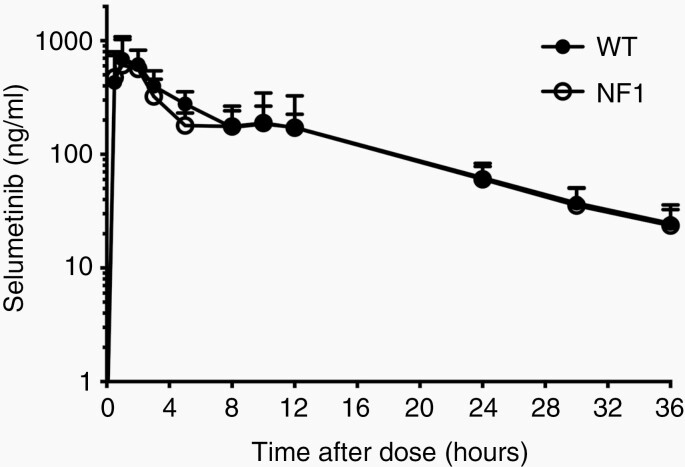
Mean plasma concentration–time profiles of selumetinib in WT and NF1 minipigs. Mean plasma concentration-time curve in WT (*n* = 8) and NF1 (*n* = 8) minipigs following a single dose of selumetinib, on a log-linear scale. Blood samples were collected immediately before dose was administered and 0.5, 1, 2, 3, 5, 8, 10, 12, 24, 30, and 36 h after administration. Plasma concentrations were determined by LC-MS/MS. Error bars represent standard error of the mean (SEM). LC-MS/MS, liquid chromatography tandem mass spectrometry; NF1, neurofibromatosis type 1; WT, wild type.

Selumetinib was rapidly absorbed in the plasma, reaching a median C_max_ of 657 ng/mL within approximately 1 h (median T_max_). Mean plasma concentration–time profiles and PK parameters showed minimal variability between individuals and genotypes (Figure 2, Supplementary Table S1), as in clinical trials. However, one WT minipig (#2038) showed low exposure and delayed T_max_ (C_max_ of 266 ng/mL; AUC of 3558 h × ng/mL, T_max_ of 5 h) (Table 1, Supplementary Table S1). In general, selumetinib plasma exposures closely matched those seen in children and adults (Table 1).^6,7,20,29^ However, with a median AUC_0-∞_ of 5361 h × ng/mL, systemic exposure was slightly higher than that of pediatric NF1 patients (2520–3855 h × ng/mL), but similar to that of healthy adults (4510–6335 h × ng/mL; Table 1).

**Table 1.** Selumetinib Plasma Exposures in Minipigs Relative to Human Patients

Group	Dose	Units	C_max_ (ng/mL)	T_max_ (hours)	T_1/2_ (hours)	AUC0→∞ (hour × ng/mL)
WT minipigs	7.4 mg/kg	Median (range)	749 (266–1270)	1.5 (0.5–5)	9.0 (6.7–12.3)	5595 (3558–9926)
NF1 minipigs	7.4 mg/kg	Median (range)	656 (294–1480)	1.1 (0.5–2)	7.8 (6.1–14.7)	4799 (4116–7927)
Pediatric patients^6^	25 mg/m^2^	Median (range)	886 (790–1200)	1.0 (1.0–2.0)	6.5 (4.7–15.8)	2520 (2341–3280)
Pediatric patients^7^	25 mg/m^2^	Median (range)	1400 (306–3570)	1.4 (1.0–4.0)	10.4 (5.4–23.1)	3855 (1780–7250)
Healthy adults^20^	75 mg	Mean (%CV)	1520 (28.5)	1.0^a^ (1.0–1.5)	13.7^b^ (5.04)	4510 (19.2)
Adult patients^29^	75 mg	Mean (range)	1207 (611–2000)	1.5^a^ (0.5–2.2)	5.33 (3.9–7.4)	6335^c^ (5260–8510)

C_max_, maximum measured selumetinib plasma concentration; T_max_, time from selumetinib administration to C_max_; T_1/2_, apparent elimination half-life; AUC_0-∞_, area under the plasma concentration–time curve from time 0 to infinity; CV, coefficient of variation.

^a^Median, range; ^b^Mean, SD; ^c^AUC_0→24 h_.

### Blood PD

**Figure 3.**
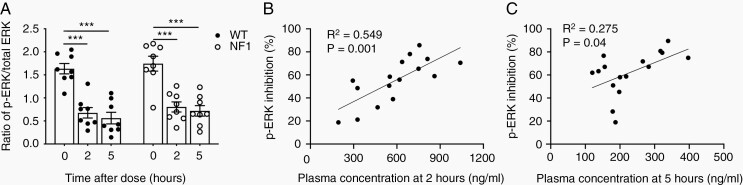
Inhibition of p-ERK in PBMCs is sustained and is significantly correlated with plasma concentration. (A) Relative expression of p-ERK in WT (*n* = 8) and NF1 (*n* = 8) PBMCs before and 2 and 5 h after selumetinib administration. Error bars represent standard error of the mean. ***P < .001, one-tailed paired *t*-test. (B) Percent inhibition of p-ERK versus Selumetinib concentration in PBMCs at 2 and 5 h (C) after Selumetinib treatment (*n* = 16) with linear regression analysis. PBMC, peripheral blood mononuclear cell; p-ERK, phosphorylated ERK; NF1, neurofibromatosis type 1; WT, wild type.

Inhibition of ERK phosphorylation was used as a pharmacodynamic biomarker for inhibition of MEK1/2 by selumetinib in ex vivo-stimulated PBMCs, a commonly used surrogate for tumor tissue in clinical trials.^6,29^ Blood samples were collected before selumetinib administration and 2 and 5 h after selumetinib administration (Figure 1A). Up to 90% inhibition of p-ERK (mean 60%) was observed from 2 to 5 h and did not differ between genotypes (Figure 3A, Supplementary Figure S1). The magnitude of p-ERK inhibition positively correlated with selumetinib plasma concentration at 2 h and 5 h (Figure 3B and C). There was no difference in p-ERK between untreated control WT and NF1 PBMCs (Figure 3A, Supplementary Figure S2).

### Tissue PK

**Figure 4.**
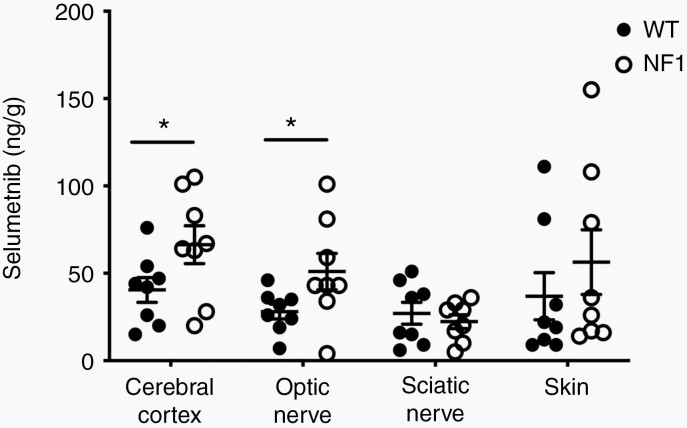
Selumetinib concentrations in minipig tissues. Selumetinib concentrations in WT and NF1 minipig tissues harvested 2 h after drug administration. Concentrations were determined by LC-MS/MS for each tissue. *P < .05, one-tailed Student’s *t*-test. LC-MS/MS, liquid chromatography tandem mass spectrometry; NF1, neurofibromatosis type 1; WT, wild type.

Tissue samples were collected 2 h after selumetinib administration (Figure 1B). This time point was chosen to allow time after T_max_ for tissue distribution and pharmacodynamic effect without significant drug clearance. Selumetinib was detected in all tissues and ranged from 4 to 155 ng/g (Figure 4, Supplementary Table S2). Median selumetinib concentrations were within the same order of magnitude between cerebral cortex (66 ng/g), optic nerve (43 ng/g), sciatic nerve (23 ng/g), and skin (34 ng/g) (Supplementary Table S2). Within each tissue, there was high interindividual variability in selumetinib exposure. Interestingly, the mean selumetinib concentration was higher in cerebral cortex (*P* = .03) and optic nerve (*P* = .03) from NF1 minipigs compared to WT minipigs (Figure 4, Supplementary Table S2).

### Tissue PD

**Figure 5.**
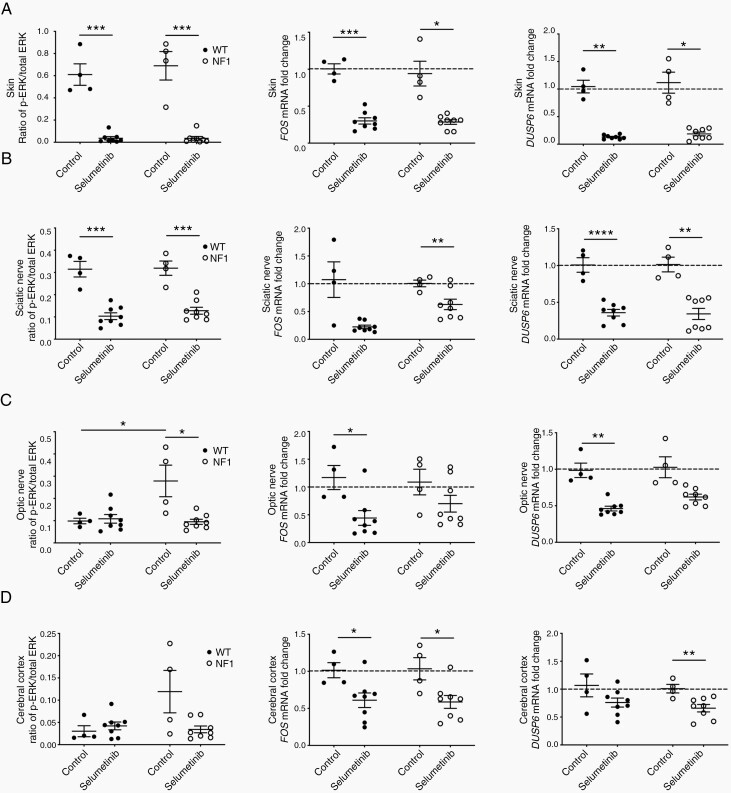
Selumetinib suppresses MAPK signaling in minipig tissues. Ratio of p-ERK to total ERK, *FOS* and *DUSP6* mRNA fold change in (A) skin, (B) sciatic nerve, (C) optic nerve, and (D) cerebral cortex from selumetinib-treated WT (*n* = 8) and NF1 (*n* = 8) minipigs and untreated WT (*n* = 4) and NF1 (*n* = 4) minipigs. *P < .05, **P < .01, ***P < .001, one-tailed Welch’s *t*-test. MAPK, mitogen-activated protein kinase; p-ERK, phosphorylated ERK; NF1, neurofibromatosis type 1; WT, wild type.

Tissue p-ERK was assessed 2 h after selumetinib administration and compared to untreated controls (Figure 1B). Inhibition of p-ERK was pronounced in the skin (mean 94% inhibition) and sciatic nerve (mean 64% inhibition) of all minipigs after selumetinib treatment, dropping to nearly undetectable levels in the skin compared to samples from untreated minipigs (Figure 5A and B). Inhibition of p-ERK was not observed in optic nerve and cerebral subcortical white matter from selumetinib-treated WT minipigs (Figure 5C and D, Supplementary Table S3). However, optic nerve from untreated NF1 minipigs showed significantly elevated p-ERK compared to WT controls (Figure 5C, Supplementary Figure S2). Selumetinib treatment resulted in inhibition of p-ERK in optic nerve from NF1 minipigs (mean 60% inhibition), effectively reducing p-ERK to WT levels (Figure 5C, Supplementary Table S3). Similarly, inhibition of p-ERK was detected in cerebral cortex from NF1 minipigs (mean 71% inhibition), but the change was not significant, likely due to low basal levels (Figure 5D, Supplementary Table S3, Supplementary Figure S3). The magnitude of selumetinib exposure in tissues did not correlate with inhibition of p-ERK in tissues, as in PBMCs (Supplementary Figure S4).

To determine whether target engagement was present in tissues with low basal levels of p-ERK like optic nerve and cerebral cortex, we evaluated the transcript expression of several Ras/MAPK pathway output genes.^30–32^ Key MAPK pathway genes *DUSP6* and *FOS* were downregulated in skin, sciatic nerve, optic nerve, and cerebral cortex of all treated minipigs, illustrating target engagement in those tissues (Figure 5A–D). Consistent with pharmacodynamic analysis of p-ERK, skin and sciatic nerve showed the most drastic decrease in target gene expression (Figure 5A and B).

To further interrogate target cell engagement, we performed immunohistochemistry (IHC) for p-ERK in minipig tissues. IHC analysis showed reduction in ERK phosphorylation in glial cells from NF1 minipig tissues treated with selumetinib (Supplementary Figures S5 and S6). Consistent with Western blot analysis, p-ERK is not easily detectable in CNS tissues from WT animals. Further, p-ERK is increased in sciatic nerve Schwann cells from untreated NF1 animals compared to WT animals, suggesting it may be more sensitive in detecting cell-specific hyperactivation of the MAPK pathway Supplementary Figure S6. Interestingly, cerebral white matter tracts from 4 of 8 treated NF1 animals did not show reduction in ERK phosphorylation by IHC Supplementary Figure S6. This could be due to incomplete drug penetrance across the blood–brain barrier (BBB) and variability in tissue sampling.

## Discussion

The success of new targeted therapies depends on their effective delivery to target tissues, and consideration of extravascular pharmacodynamics is crucial when making predictions regarding their therapeutic application.^33^ This is particularly important in NF1 as the blood–nerve or BBB can be a major impediment to adequate drug delivery in peripheral nerve sheath tumors, optic nerve gliomas, and brain tumors common in NF1 patients.^34,35^ Evaluation of PK and PD in NF1-relevant tissues has been a challenge because these invasive studies are not generally feasible in human patients and differences in metabolism, organ size, and physiology cause difficulty translating murine models to humans. In this study, we show that NF1 minipigs are a valuable predictive model for preclinical evaluation of new targeted therapies by demonstrating the pharmacodynamic effects of selumetinib in key tissues relevant to NF1.

At doses that correspond to human therapeutic equivalent doses, oral selumetinib resulted in rapid absorption in the plasma and sustained inhibition of p-ERK in activated PBMCs. The plasma PK and PD profiles of selumetinib in minipigs were similar to those reported in other preclinical and clinical studies of MEK inhibitors, demonstrating the translational value of the minipig.^6,7,20,29,36^ The magnitude of p-ERK inhibition was positively correlated with selumetinib plasma exposure in PBMCs at 2 and 5 h, as seen in adult patients with advanced cancer.^29^ Interestingly, this correlation was not observed in tissues, suggesting that tissue PK may not be a relevant indicator of target inhibition or drug efficacy in tissues, or that the concentrations attained at these sites are higher than necessary to inhibit MAPK activity.

Selumetinib was detected in all tissues from WT and NF1 minipigs within 2 h of drug administration, indicating efficient distribution of selumetinib into the extravascular space and across the BBB. Other preclinical studies in healthy mice and rats have shown a diminished ability of selumetinib to penetrate the brain compared to other MEK inhibitors, suggesting poor penetration of selumetinib against an intact BBB.^25,35,37,38^ The median brain–plasma ratio of selumetinib in minipigs was 0.1, which is similar to that seen in a preclinical study with a panel of MEK inhibitors in healthy mice.^37^ Nonetheless, selumetinib was detected in both the optic nerve and the cerebral cortex and showed a pharmacodynamic effect in optic nerve from NF1 minipigs after a single dose. In agreement with this data, selumetinib shows activity in pediatric patients with NF1-associated low-grade glioma.^5,7^ However, adverse events are common and some patients progress despite treatment.^5,7^ This could be due to individual differences in tissue penetration or acquired efflux transporter-mediated resistance in certain patients. Future studies are needed to evaluate the brain penetrance and efficacy of selumetinib as well as other NF1-targeted therapies. The NF1 minipig develops optic pathway glioma and offers an ideal platform for these studies.^13^

Interestingly, although plasma exposure was similar between WT and NF1 minipigs, selumetinib concentrations were higher in CNS tissues from NF1 minipigs. It is possible that a germline defect in *NF1* expression affects selumetinib uptake in certain tissues. In fact, one clinical trial reported increased drug exposure and dose-limiting toxicities specifically in NF1 children compared to patients with other advanced cancers, although this outcome has not been reported with MEK inhibitors.^39^ To the best of our knowledge, selumetinib entry and distribution in CNS tissue has not been evaluated in other preclinical models of NF1 and compared to WT. However, there is evidence that *Nf1* loss in mouse oligodendrocytes causes defects in the BBB, which could explain the increased selumetinib exposure in CNS tissues from NF1 compared to WT animals.^40^ It is also conceivable that *NF1* expression may affect astrocytes that play a significant role in maintaining the BBB. Astrocytes, neurons, and cerebral microvasculature together act as functional “neurovascular units” to form the BBB, where astrocytes are the key link in these units, acting as crucial intermediaries in intercellular signaling.^41^ Perivascular astrocytes communicate with both synapses and blood vessel pericytes and vascular smooth muscle as well as other astrocytes via gap junctions and through the release of ATP.^42^ It will be important to evaluate the steady state tissue distribution of selumetinib in WT and NF1 minipigs to better understand the implications of these results.

Selumetinib has been shown to reduce p-ERK in patient PBMCs and paired tumor biopsies, but these pharmacodynamic analyses have not been conducted in clinically relevant non-tumor tissues from NF1 patients.^29,36,43^ In our study, p-ERK was measurable in all tissues from both WT and NF1 minipig untreated controls. At baseline, skin from all untreated minipigs showed high p-ERK, sciatic nerve showed an intermediate level of p-ERK, and CNS tissues had relatively low p-ERK levels. Basal p-ERK levels were generally similar between genotypes despite the *NF1* loss-of-function mutation in NF1 minipigs, with the exception of optic nerve. Similarly, basal levels of *FOS* and *DUSP6* were not significantly different between genotypes. This observation suggests that in some tissues, loss of one functional allele of NF1 is sufficient to cause hyperactive Ras signaling, while other tissues only require one WT copy of NF1 for sufficient neurofibromin activity. Enhanced activity of other Ras-GTPase activating proteins (GAPs), feedback inhibitors of the Ras pathway such as Sprouty proteins and dual specificity phosphatases (DUSPs), or differences in upstream pathway activators may also help compensate for decreased neurofibromin expression in certain tissues.^44,45^ Strikingly, optic nerve from NF1 minipigs treated with selumetinib showed a significant reduction in p-ERK to WT levels. Similarly, *FOS* and *DUSP6* gene expression were reduced, but not significantly. This result suggests that selumetinib is able to cross the BBB at sufficient levels to normalize effects on hyperactive MAPK signaling in some CNS tissues at clinically achievable plasma concentrations.

Notably, a single dose of selumetinib reduced p-ERK, *FOS* and *DUSP6* levels in the skin to nearly undetectable levels. This is particularly striking given the high basal levels of MAPK signaling in the skin of both WT and NF1 minipigs. Given this result, it is not surprising that dermatologic toxicities are the most common adverse events associated with MEK inhibitors, including selumetinib.^46–48^ These and other adverse events like gastrointestinal and cardiovascular toxic effects can severely diminish quality of life, resulting in lack of adherence to treatment and clinical trial failure.^48^ Refinements in future trials with MEK inhibitors such as more conservative starting doses, intermittent dosing, or alternative routes of administration may result in sustained normalization of Ras signaling without severe toxicities in non-target tissues. Given the relatively short half-life of selumetinib, alternative dosing schedules may reduce toxicity without lowering intracranial drug concentrations. This could be evaluated in a longitudinal study using the NF1 minipig model. Additionally, selumetinib in combination with other targeted agents may result in synergistic or additive responses due to targeting different pathways, thereby reducing the dose required for therapeutic efficacy without intolerable side effects.^49,50^ Inhibition of Ras/MAPK signaling results in relief of negative feedback that can result in pathway re-activation, therefore fine-tuning dose and schedule and introducing drug combinations may be more effective at preventing tumor progression while potentially mitigating side effects. While outside of the scope of this study, evaluating inhibition of MAPK signaling at later time points and in combination with other therapies in NF1 minipigs could be useful for evaluating mechanisms of acquired resistance as well as guiding alternative dosing schedules that would require much longer to develop in human patients.^51^

Our study suggests that tissue pharmacodynamics could be important for guiding therapeutic dosing strategies, and that performing these studies in preclinical disease models like the NF1 minipig may improve success rates in clinical trials. However, it may be difficult to make conclusions in some tissue types because regulation of Ras-MAPK signaling varies between tissues, thus even if molecular therapies reach their target, they may be more or less able to reduce signaling to ERK for reasons that are unclear and warrant further study. For example, other Ras-GAPs and feedback inhibition with DUSPs may be more relevant in certain tumors and tissues.^45^

Future preclinical studies in the NF1 minipig will evaluate selumetinib tolerability and toxicity, tumor response and inhibition of p-ERK in cutaneous neurofibromas, and tissue pharmacology of selumetinib in combination with other targeted therapies. The results of this study ultimately provide further evidence that the NF1 minipig represents a valuable preclinical disease model and demonstrate the importance of conducting safety and efficacy studies in large animal models of human diseases to better inform clinical trials.

## Supplementary Material

vdab020_suppl_Supplementary_Figure_S1

vdab020_suppl_Supplementary_Figure_S2

vdab020_suppl_Supplementary_Figure_S3

vdab020_suppl_Supplementary_Figure_S4

vdab020_suppl_Supplementary_Figure_S5

vdab020_suppl_Supplementary_Figure_S6

vdab020_suppl_Supplementary_Table_S1

vdab020_suppl_Supplementary_Table_S2

vdab020_suppl_Supplementary_Table_S3

## Data Availability

The data that support the findings of this study are available from the corresponding author, A.L.W., upon reasonable request.

## Notes

**Authorship Statement** Concept of study: S.H.O., C.L.M., D.A.L., and A.L.W. Experimental design: S.H.O., M.N.K., P.D.S., B.C.W., C.L.M., D.A.L., and A.L.W. Data acquisition: S.H.O., A.W.C., D.J.D., B.R.T., M.N.K., J.F., O.D., and A.L.W. Data analysis: S.H.O., M.N.K., W.R.B., B.C.W., C.L.M., D.A.L., and A.L.W. Draft of manuscript: S.H.O. Careful review of manuscript: A.W.C., D.J.D., B.R.T., M.N.K., P.D.S., W.R.B., O.D., B.C.W., C.L.M., D.A.L., and A.L.W.

## Notes

**Conflict of interest statement**. A.L.W., A.W.C., D.J.D., B.R.T., and D.A.L. are employees and/or shareholders of Recombinetics Inc. C.L.M. is a consultant to Recombinetics Inc. DAL is the co-founder and co-owner of several biotechnology companies including NeoClone Biotechnologies, Inc., Discovery Genomics, Inc. (recently acquired by Immusoft, Inc.), B-MoGen Biotechnologies, Inc. (recently acquired by the Biotechne corporation), and Luminary Therapeutics, Inc. He consults for Genentech, Inc., which is funding some of his research. D.A.L. holds equity in, is a scientific advisor to, and serves on the Board of Directors of Recombinetics, a genome-editing company. The business of all these companies is unrelated to the contents of this manuscript, with the exception of Recombinetics, which developed the animal model used in these studies. P.D.S. and O.D. are employees of AstraZeneca PLC. S.H.O., M.N.K., J.F., and B.C.W. have no disclosures.
