# Supplementary material for: Selumetinib normalizes Ras/MAPK signaling in clinically relevant neurofibromatosis type 1 minipig tissues in vivo
Source: Neurooncol Adv. 2021 Feb 10;3(1):vdab020. doi: 10.1093/noajnl/vdab020 (PMC8095338; doi:10.1093/noajnl/vdab020)
Supplement: vdab020_suppl_Supplementary_Figure_S1 [file vdab020_suppl_supplementary_figure_s1.docx]

Supplementary Figure S1. Virtual blot view of Wes data showing p-ERK and ERK in peripheral blood mononuclear cells from eight WT and eight NF1 animals at 0, 2, and 5 hours after selumetinib administration. Lanes were compiled from multiple runs. Sample key is presented on the left side.
