# Supplementary material for: Selumetinib normalizes Ras/MAPK signaling in clinically relevant neurofibromatosis type 1 minipig tissues in vivo
Source: Neurooncol Adv. 2021 Feb 10;3(1):vdab020. doi: 10.1093/noajnl/vdab020 (PMC8095338; doi:10.1093/noajnl/vdab020)
Supplement: vdab020_suppl_Supplementary_Figure_S2 [file vdab020_suppl_supplementary_figure_s2.docx]

Figure S2. Mean p-ERK in tissues from untreated NF1 minipigs relative to untreated WT minipigs. Values are expressed as a ratio of mean NF1/WT p-ERK expression normalized to total ERK.
