# Supplementary material for: Selumetinib normalizes Ras/MAPK signaling in clinically relevant neurofibromatosis type 1 minipig tissues in vivo
Source: Neurooncol Adv. 2021 Feb 10;3(1):vdab020. doi: 10.1093/noajnl/vdab020 (PMC8095338; doi:10.1093/noajnl/vdab020)
Supplement: vdab020_suppl_Supplementary_Figure_S3 [file vdab020_suppl_supplementary_figure_s3.docx]

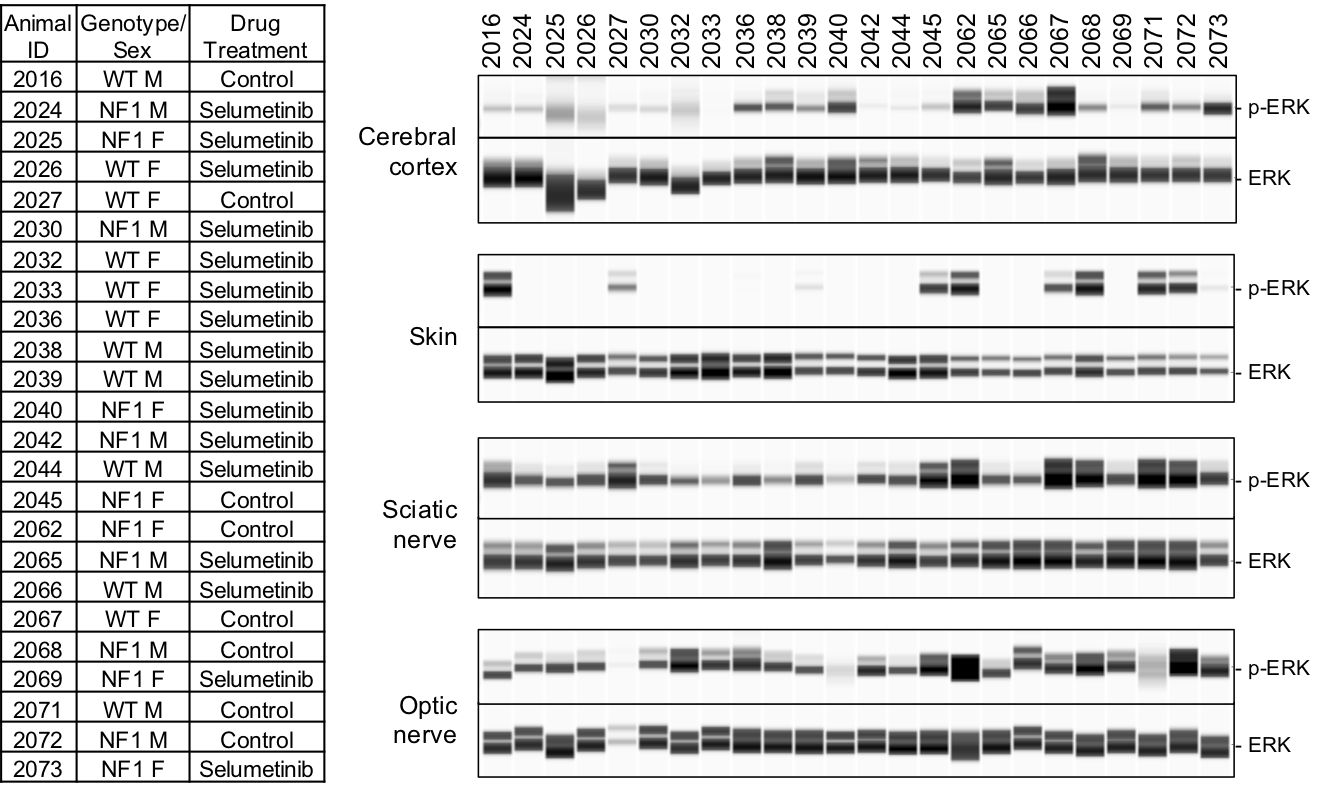


Supplementary Figure S3. Virtual blot view of Wes data showing p-ERK and ERK in cerebral cortex, skin, sciatic nerve, and optic nerve from selumetinib-treated (n=8) and control (n=4) WT and NF1 animals. Sample key is presented on the left side.
