# Supplementary material for: Selumetinib normalizes Ras/MAPK signaling in clinically relevant neurofibromatosis type 1 minipig tissues in vivo
Source: Neurooncol Adv. 2021 Feb 10;3(1):vdab020. doi: 10.1093/noajnl/vdab020 (PMC8095338; doi:10.1093/noajnl/vdab020)
Supplement: vdab020_suppl_Supplementary_Figure_S4 [file vdab020_suppl_supplementary_figure_s4.docx]

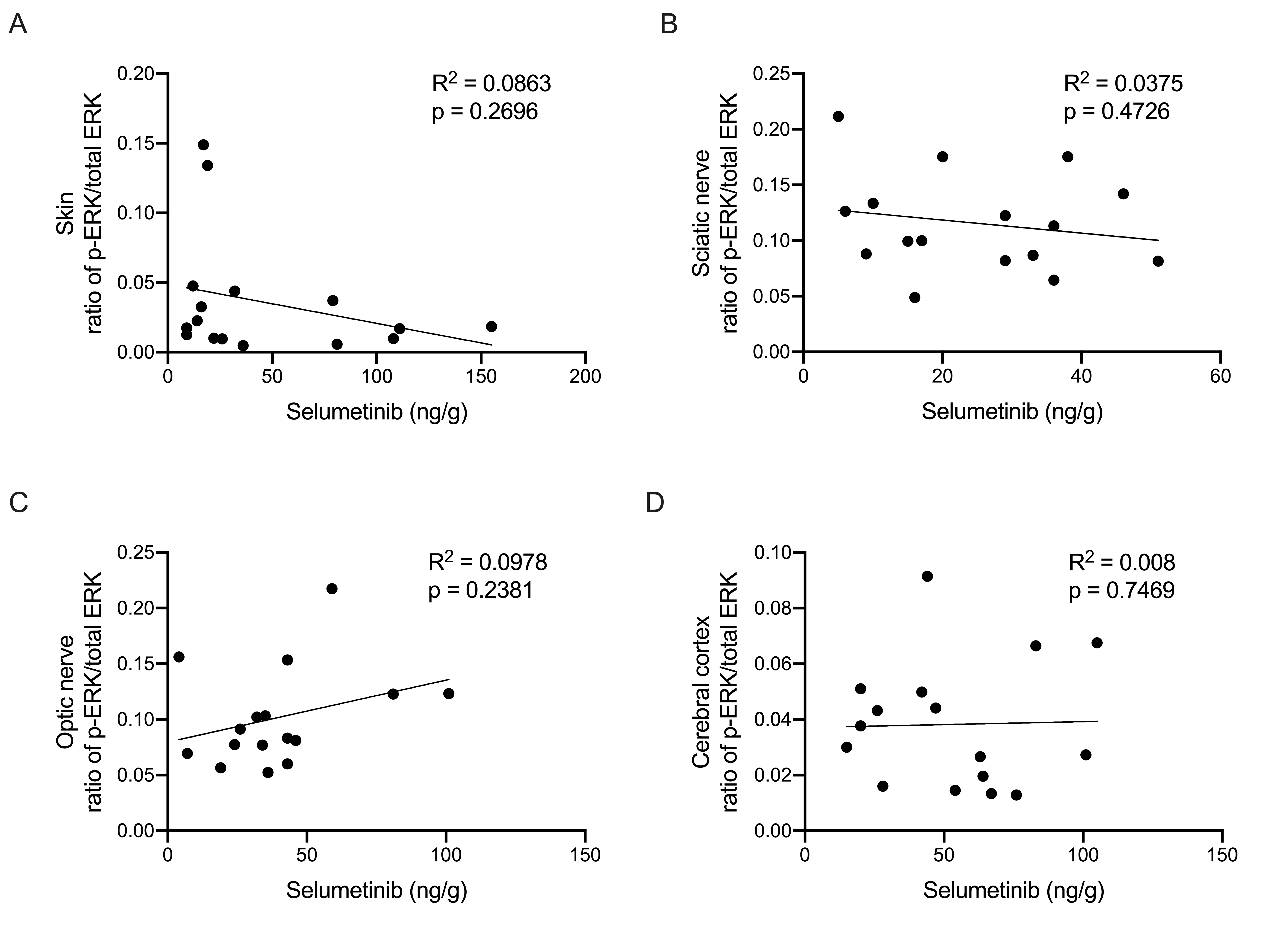


Supplementary Figure S4. Relationship of selumetinib tissue concentration and p-ERK expression. Relative p-ERK expression versus selumetinib concentration in (A) skin, (B) sciatic nerve, (C) optic nerve, and (D) cerebral cortex from WT and NF1 minipigs (n=16) with linear regression analysis.
