# Supplementary material for: Selumetinib normalizes Ras/MAPK signaling in clinically relevant neurofibromatosis type 1 minipig tissues in vivo
Source: Neurooncol Adv. 2021 Feb 10;3(1):vdab020. doi: 10.1093/noajnl/vdab020 (PMC8095338; doi:10.1093/noajnl/vdab020)
Supplement: vdab020_suppl_Supplementary_Figure_S5 [file vdab020_suppl_supplementary_figure_s5.docx]

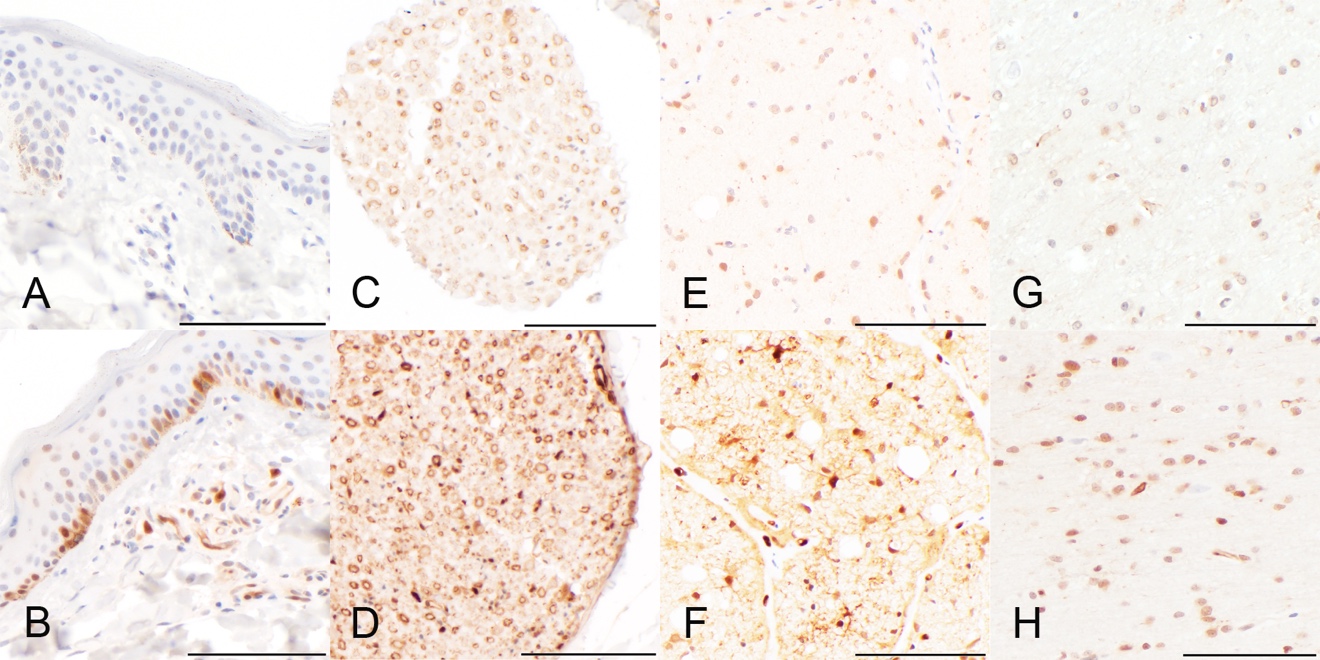


Figure S5. Immunohistochemical staining for p-ERK of NF1 minipigs with or without selumetinib administration. Representative histologic sections (scale=100 microns, A-H) of skin (A-treated, B-untreated)- labels basal epithelial cells and subcutaneous nerves; cross sections of sciatic nerve (C-treated, D-untreated) - labels Schwann cells; cross sections of optic nerve (E-untreated, F-treated) - labels astrocytes within fascicles subdivided by pial septae; and highlights subcortical white matter glia within regions of the neocortex (G-treated, H-untreated).
