# Supplementary material for: Selumetinib normalizes Ras/MAPK signaling in clinically relevant neurofibromatosis type 1 minipig tissues in vivo
Source: Neurooncol Adv. 2021 Feb 10;3(1):vdab020. doi: 10.1093/noajnl/vdab020 (PMC8095338; doi:10.1093/noajnl/vdab020)
Supplement: vdab020_suppl_Supplementary_Figure_S6 [file vdab020_suppl_supplementary_figure_s6.docx]

Supplementary Figure S6. Heat map of Immunohistochemical analysis of p-ERK of WT and NF1 minipig tissues with or without selumetinib administration. Tissue staining was scored as no (0), low (1) (1-10 cells), moderate (2) (11-20 cells) and high (3) (>20 cells) in examined tissues.
