# Supplementary material for: Selumetinib normalizes Ras/MAPK signaling in clinically relevant neurofibromatosis type 1 minipig tissues in vivo
Source: Neurooncol Adv. 2021 Feb 10;3(1):vdab020. doi: 10.1093/noajnl/vdab020 (PMC8095338; doi:10.1093/noajnl/vdab020)
Supplement: vdab020_suppl_Supplementary_Table_S1 [file vdab020_suppl_supplementary_table_s1.docx]

| **Genotype** | **Animal ID** | **C_max_**  **(ng/ml)** | **T_max_**  **(hour)** | **T_1/2_**  **(hour)** | **K_el_**  **(hour^-1^)** | **C_last_**  **(mg/l)** | **AUC_0🡪∞_**  **(hour*ng/ml)** |
| --- | --- | --- | --- | --- | --- | --- | --- |
| NF1 F | 2025 | 812 | 2.0 | 7.5 | 0.1 | 8.3 | 3556 |
| NF1 F | 2040 | 678 | 1.1 | 7.6 | 0.1 | 31.8 | 5896 |
| NF1 F | 2069 | 633 | 0.5 | 8.0 | 0.1 | 22.2 | 5255 |
| NF1 F | 2073 | 571 | 0.5 | 8.2 | 0.1 | 20.1 | 4287 |
| NF1 M | 2024 | 294 | 2.0 | 14.7 | 0.0 | 31.3 | 4116 |
| NF1 M | 2030 | 766 | 1.0 | 10.6 | 0.1 | 34.9 | 6092 |
| NF1 M | 2042 | 547 | 2.0 | 6.1 | 0.1 | 11.8 | 4344 |
| NF1 M | 2065 | 1480 | 1.1 | 7.3 | 0.1 | 21.3 | 7928 |
| WT F | 2026 | 636 | 2.0 | 7.1 | 0.1 | 13.9 | 3675 |
| WT F | 2032 | 573 | 2.0 | 10.1 | 0.1 | 28.5 | 5722 |
| WT F | 2033 | 1040 | 1.1 | 6.7 | 0.1 | 13.0 | 5468 |
| WT F | 2036 | 1020 | 0.5 | 7.8 | 0.1 | 20.3 | 5843 |
| WT M | 2038 | 266 | 5.0 | 12.3 | 0.1 | 25.0 | 3558 |
| WT M | 2039 | 621 | 0.5 | 6.7 | 0.1 | 38.6 | 9926 |
| WT M | 2044 | 861 | 2.1 | 10.6 | 0.1 | 28.2 | 5995 |
| WT M | 2066 | 1270 | 1.0 | 11.8 | 0.1 | 23.1 | 5040 |
| Median  (range) |  | 657  (266-1480) | 1.1  (0.5-5) | 7.9  (6.1-14.7) | 0.1  (0.05-0.11) | 22.7  (8.3-38.6) | 5361  (3556-9926) |

**Supplementary Table S1: ﻿Individual selumetinib plasma pharmacokinetic parameters.** Abbreviations: C_max_ = maximum measured drug plasma concentration, T_max_ = time from drug administration to C_max_, T_1/2_ = apparent elimination half-life, AUC_0-∞_ = area under the plasma concentration–time curve from time 0 to infinity, K_el_ = Elimination rate constant, C_last_ = clearance.
