# Supplementary material for: Selumetinib normalizes Ras/MAPK signaling in clinically relevant neurofibromatosis type 1 minipig tissues in vivo
Source: Neurooncol Adv. 2021 Feb 10;3(1):vdab020. doi: 10.1093/noajnl/vdab020 (PMC8095338; doi:10.1093/noajnl/vdab020)
Supplement: vdab020_suppl_Supplementary_Table_S2 [file vdab020_suppl_supplementary_table_s2.docx]

| **Genotype/sex** | **Animal ID** | **Selumetinib concentration in tissues (ng/g)** | | | |
| --- | --- | --- | --- | --- | --- |
|  |  | **Cerebral**  **cortex** | **Optic**  **nerve** | **Sciatic**  **nerve** | **Skin** |
| NF1/F | 2025 | 63 | 43 | 20 | 36 |
| NF1/F | 2040 | 105 | 81 | 10 | 155 |
| NF1/F | 2069 | 101 | 101 | 17 | 14 |
| NF1/F | 2073 | 20 | 4 | 5 | 17 |
| NF1/M | 2024 | 28 | 43 | 36 | 108 |
| NF1/M | 2030 | 64 | 34 | 33 | 26 |
| NF1/M | 2042 | 67 | 43 | 29 | 16 |
| NF1/M | 2065 | 83 | 59 | 29 | 79 |
| *Median (NF1)* |  | 66 | 43 | 25 | 56 |
| *Range (NF1)* |  | (20-105) | (4-101) | (5-36) | (14-155) |
| WT/F | 2026 | 26 | 24 | 46 | 22 |
| WT/F | 2032 | 42 | 26 | 38 | 111 |
| WT/F | 2033 | 76 | 19 | 36 | 81 |
| WT/F | 2036 | 47 | 46 | 16 | 12 |
| WT/M | 2038 | 20 | 35 | 6 | 9 |
| WT/M | 2039 | 15 | 7 | 9 | 19 |
| WT/M | 2044 | 54 | 32 | 51 | 9 |
| WT/M | 2066 | 44 | 36 | 15 | 32 |
| *Median (WT)* |  | 43 | 29 | 26 | 21 |
| *Range (WT)* |  | (15-76) | (7-46) | (6-51) | (9-111) |
| *Median (All)* |  | 51 | 36 | 25 | 24 |
| *Range (All)* |  | (15-105) | (4-101) | (5-51) | (9-155) |

**Supplementary Table S2. Selumetinib concentrations in minipig tissues.** Abbreviations: F = female, M = male.
