# Supplementary material for: Selumetinib normalizes Ras/MAPK signaling in clinically relevant neurofibromatosis type 1 minipig tissues in vivo
Source: Neurooncol Adv. 2021 Feb 10;3(1):vdab020. doi: 10.1093/noajnl/vdab020 (PMC8095338; doi:10.1093/noajnl/vdab020)
Supplement: vdab020_suppl_Supplementary_Table_S3 [file vdab020_suppl_supplementary_table_s3.docx]

| **Genotype** | **Tissue** | **p-ERK**  **Inhibition (%)** |
| --- | --- | --- |
| NF1 | Skin | 95 |
| WT | Skin | 94 |
| NF1 | Sciatic nerve | 60 |
| WT | Sciatic nerve | 67 |
| NF1 | Optic nerve | 60 |
| WT | Optic nerve | ND |
| NF1 | Cerebral cortex | 71 |
| WT | Cerebral cortex | ND |

**Supplementary Table S3. Percent inhibition of p-ERK in WT and NF1 minipig tissues.** % Inhibition = [(untreated - treated)/(untreated)] *100. Abbreviations: ND = Not detected
